# Soil microbial properties of subalpine steppe soils at different grazing intensities in the Chinese Altai Mountains

**DOI:** 10.1038/s41598-021-81120-y

**Published:** 2021-01-18

**Authors:** Sven Goenster-Jordan, Mariko Ingold, Ramia Jannoura, Andreas Buerkert, Rainer Georg Joergensen

**Affiliations:** 1 Organic Plant Production and Agroecosystems Research in the Tropics and Subtropics, University of Kassel, Steinstr. 19, 37123 Witzenhausen, Germany; 2 Soil Biology and Plant Nutrition, University of Kassel, Nordbahnhofstr. 1a, 37213 Witzenhausen, Germany

**Keywords:** Microbiology, Environmental sciences, Agroecology, Ecosystem services, Grassland ecology

## Abstract

Long-term provision of ecosystem services by grasslands is threatened by increasing stocking densities. The functions of grassland ecosystems depend on a mutual relationship between aboveground and belowground biota. While the effects of increasing stocking density on plant biomass are well studied, little is known about its impact on soil microbial properties. To fill this knowledge gap a grazing experiment was conducted on a summer pasture in the Chinese Altai Mountains during the summers of 2014 and 2015 using a randomized block design with stocking densities of 0, 8, 16, and 24 sheep ha^−1^ replicated four times. After two summer grazing periods (each 56 days), topsoil samples (1–7 cm) were taken in September 2015 and analyzed for major physical, chemical, and microbial soil properties. Except for the metabolic quotient (*q*CO_2_; p < 0.05), the examined soil properties remained unaffected by the increasing stocking densities, likely due to high spatial variability. The *q*CO_2_ declined from 13.5 mg CO_2_–C g^−1^ microbial biomass C d^−1^ at zero grazing to 12.2 mg CO_2_–C g^−1^ microbial biomass C d^−1^ at a stocking density of 24 sheep ha^−1^. Low values of *q*CO_2_ indicate an aged and dormant microbial community that diverts less soil organic carbon (SOC) to catabolic processes within their cells, characteristic for C limiting conditions. The aboveground biomass affected by grazing intensity correlated positively with SOC (r_s_ = 0.60, p = 0.015) and ergosterol (r_s_ = 0.76, p = 0.001) pointing indirectly to the effect of stocking density. Additionally to the relatively high values of *q*CO_2_, highest values of SOC (39.2 mg g^−1^ soil), ergosterol (6.01 µg g^−1^ soil), and basal respiration (10.7 µg g^−1^ soil d^−1^) were observed at a stocking density of 8 sheep ha^−1^ indicating that a low grazing intensity is recommendable to avoid soil degradation.

## Introduction

With a total area of about 10 million km^2^, which represents about 7% of the global terrestrial surface, the Palaearctic steppe biome is the largest continuous rangeland in the world^1^. About 3 million km^2^ of this rangeland is located in Northwest China, accounting for 40% of the national territory of the People’s Republic, and is generally dominated by grasses and forbs (grassland)^2,3^. This grassland provides ecosystem services that are essential for the livelihood of the majority of the region's population^4,5^. These ecosystem services comprise provisioning services such as forage and livestock production, and fresh water supply, as well as regulating services such as water erosion control and nutrient retention^6^. However, the grassland of the region is subjected to severe land degradation that limits the provision of ecosystem services, primarily caused by overgrazing^7,8^. Overgrazing has increased in recent decades due to the significant increase in livestock numbers^9^ in response to the rapidly rising demand for livestock products such as milk and meat^10^. In Xinjiang, for example, 70% of the total rangeland is reportedly overgrazed^7^, whereby the actual stocking capacity exceeds the theoretical one by a factor of 2.5, as similarly observed throughout northwest China^11^.

The ecosystem services of grasslands largely depend on the mutual relationship between aboveground and belowground biota (i.e. between plants and soil organisms)^12^, in which microbial communities have a pivotal role owing to their high number and biomass^13–15^. Soil microorganisms can affect plant productivity and diversity in grassland^16^, mainly due to their pathogenicity and their influence on the availability of nutrients through mineralization^17^. Plant biomass and diversity in turn affect soil microbial biomass, community composition and activity mainly through root-derived organic inputs in form of root biomass and exudates^18–20^. These above- and belowground relationships are affected by grazing livestock through consumption of plant biomass, excretion input and treading, whereby the effects’ magnitude are modulated by stocking density^21^.

Recent meta-studies have shown that increasing grazing intensities in China generally have a negative impact not only on above—but also on belowground plant biomass^22,23^. This can lead to a reduction in plant C allocation and root excretion patterns, having a negative effect on microbial biomass and activity^24^. For a typical steppe in Inner Mongolia in China, microbial biomass carbon (MBC) and soil organic carbon (SOC) correlated, i.e., both SOC and MBC increased under light grazing intensities compared to higher ones^25^. For the same region, a likewise trend was reported not only for MBC but also for microbial activity indicators such as basal respiration and the metabolic quotient *q*CO_2_^26^. Furthermore, grazing can change the spatial distribution of resources available for soil microorganisms by returning plant biomass in the form of feces and urine to soil^21,24^. Depending on stocking densities, the deposition of excrements thereby leads to a heterogeneous concentration of labile carbon and nutrients in the topsoil (0–10 cm) stimulating the soil microbial biomass and its activity at local spots^23,27^.

The mechanical impact of livestock hooves modifies soil aggregates and bulk density affecting the community structure and biomass of soil microorganisms^21^. Increased trampling can contribute to a reduction of microbial biomass and a shift of the microbial community structure from a fungi-dominated to a bacteria-dominated one. This has been observed in Swiss subalpine meadows^28^ and in meadow steppes of Inner Mongolia, China^29^. The effect of grazing on plant biomass is well investigated^23^ as is the link between plants and soil microorganisms which plays a pivotal role in nutrient cycling and plant productivity^12^. In contrast, much less is known about the impact of different stocking densities on biological soil properties. Biological soil properties respond sensitively and rapidly to environmental changes^30^, possibly qualifying them as indicators for changes in soil quality caused by variations in grazing management.

In view of the above-mentioned relationships, the present study tests the hypothesis that grazing mainly affects microbial properties of the topsoil within a few grazing periods. Thereby, it is assumed that different grazing intensities affect primarily the level of C and nutrient inputs into the soil with respective effects on microbial biomass and activity. Based on a previous two-year grazing experiment that showed a response of aboveground biomass (AGB) to different stocking densities^31^, the specific objectives of this study were to (i) determine the effect of different stocking densities on soil microbial biomass and activity besides major physical and chemical soil properties; (ii) identify relationships between soil properties (microbial biomass and activity as well as physical and chemical parameters) and aboveground biomass on a subalpine steppe in the Altai Mountains, Xinjiang, China.

## Materials and methods

### Study area

The experimental site is situated at Akbulak (47°12′23.62" N, 90°14′58.20" E, 2400 m a.s.l.) in the Chinese Altai Mountain range north of Qinghe, Qinghe County, Xinjiang Uyghur Autonomous Region of China. The climate is characterized by high temperature fluctuations during the long and cold winters, and short summers of moderate temperature^32^. Despite its location near the dry Dzungarian Gobi with a semi-arid to arid climate, in higher altitudes the area receives substantial rainfall in both summer and winter. The long-term average annual rainfall amount over 50 years in Qinghe was 174 mm, with average minimum temperatures of − 34 °C in January and average maximum temperatures of 24 °C in July (based on data from 1958 to 2007 of the meteorological station in Qinghe, 46°40′28" N, 90°22′59" E, 1253 m a.s.l.;^33^). At the experimental site, average air temperature was − 1.1 °C (2013–2014) and mean annual precipitation amounted to 211 mm across the three years (2012–2014) with a high interannual variation ranging from 133 mm (in 2014) to 314 mm (in 2012). Since, millennia the area is used as a high altitude summer pasture from early July until early September depending on the actual weather conditions^33^. The hilly grassland, in which the experimental area was located, is situated near the tree line and is classified as an upper montane to subalpine meadow steppe with a vegetation cover of various grasses and forbs of high small-scale variability^31^. The vegetation comprised the *Agropyron cristatum* community, the *Festuca ovina*, *Festuca altaica* and *Phlomis tuberosa* community, and the *Juniperus sabina* and *Larix sibirica* community^33^. The most important species were *Helictotrichon pubescens* (Huds.) Schult. & Schult.f., *Festuca valesiaca* Schleich ex. Gaudin, *Alchemilla pinguis* Juz., *Cerastium cerastoides* (L.) Britton, *Dracocephalum nutans* L. and *Galium verum* L.^31^. The experimental site was oriented to the north and declined from north to south, with the upper third of the site being flat and the lower two thirds having a slope of 5 to 10%^31^.

### Experimental design and sampling

The experiment was conducted in a large exclosure (350 × 150 m^2^), which was established in 2012 to keep out livestock. The exclosure was subdivided by portable fences into 16 paddocks of 2500 m^2^ each. In 2012 and 2013 a test pre-treatment was conducted to test the herbage allowance and to monitor the spatial and temporal variability of the vegetation cover^31^. In the main experimental phase during the summer 2014 and summer 2015, the treatments and replicates were laid out in a randomized complete block design (n = 4). Based on the available biomass, stocking densities were set to 0, 8, 16, and 24 sheep ha^−1^ for a grazing period of 56 days^31^. Adult fat-rumped sheep (rams and ewes) of the Altai breed typical for western Xinjiang were bought each spring from nearby herder families. The animals had an average weight of 35.5 kg in 2014 and 26.1 kg in 2015, and were provided with ad libitum access to drinking water and mineral licks^31^.

As reported by Lv et al.^31^, AGB was recorded in intervals of about 20 days, starting in early July until beginning of September, using four 0.25 m^2^ quadrats in each paddock. The biomass was clipped at 1 cm height within the grazed paddocks to mimic the animal’s grazing impact^31^. By the end of the experiment in September 2015, six subsamples were taken from each plot at a depth of 1 to 7 cm from the soil surface (n = 96). Per subsample, 117.81 cm^3^ of soil material was collected using a 6 cm long sampling tube with an inner diameter of 5 cm. Sampling occurred after removal of vegetation cover in a raster of two parallel three-point lines with an orthogonal distance between the points of 10 to 15 m followed by storage in polypropylene bags at 4 °C until analyses.

### Analyses of soil samples

Prior to analyses between December 2016 and February 2017, all soil samples were sieved to < 2 mm particle size and visible plant roots were carefully removed. Texture was determined by wet sieving for the sand fraction (> 63 µm) and by gravitational sedimentation (pipette method) for the silt and clay fractions after removing organic material with 10% H_2_O_2_^34^. Carbonate (CaCO_3_), electrical conductivity (EC), and pH were analyzed in air-dried soil. Carbonate was measured gas-volumetrically after the addition of 10% HCl using a Scheibler apparatus (Calcimeter Bernard, Prolabo, Paris, France;^35^). Electrical conductivity was determined using a digital conductivity meter (3430, GHM Messtechnik GmbH, Regenstauf, Germany) at a soil/water ratio of 1/2.5. Soil pH was measured at a soil/water ratio of 1/2.5 by a glass electrode (ProLab 1000, SI Analytics GmbH, Mainz, Germany). For the analysis of total C and N by a Vario MAX CN analyzer (Elementar Analysensysteme GmbH, Langenselbold, Germany), soil was oven dried at 105 °C for 24 h and ground with a ball mill. Soil organic carbon (SOC) was calculated by subtracting carbonate C from total C. The water holding capacity (WHC) of the soil was determined by the amount of water held in the soil sample relative to the dry weight of the sample (105 °C; 24 h).

For measuring MBC and microbial biomass nitrogen (MBN), as well as basal respiration, samples were adjusted to 50% of WHC and pre-incubated at 22 °C for 7 days. MBC and MBN were determined by chloroform fumigation extraction^36,37^ including a pre-extraction step^38^. For pre-extraction, 30 g of soil and 80 ml 0.05 M K_2_SO_4_ were horizontally shaken for 30 min at 200 rev min^−1^ and centrifuged for 10 min at 2000 g. Then one aliquot of 10 g was fumigated for 24 h with ethanol-free CHCl_3_ and extracted thereafter with 40 ml of 0.5 M K_2_SO_4_. The second non-fumigated aliquot was directly extracted in the same way. Organic C and total N in the K_2_SO_4_ extracts were determined using a CN analyzer (Multi N/C 2100S, Analytik Jena AG, Germany). MBC and MBN were calculated as the difference between organic C or total N from fumigated soils and non-fumigated soils (C or N extracted from fumigated soils minus C or N extracted from non-fumigated soils), respectively, divided by a constant. The constant used to calculate MBC according to Wu et al.^39^ was k_EC_ = 0.45 and for MBN k_EN_ = 0.54^36^.

Basal respiration of 50 g of the pre-incubated soil was estimated by trapping the evolving CO_2_ in 0.5 M NaOH over a period of 7 days at 22 °C within a closed incubation system. By back-titration of the unreacted NaOH with 0.5 M HCl, after the precipitation of C with saturated BaCl_2_ solution, the amount of evolved CO_2_ was calculated from the volume of used HCl. The metabolic quotient *q*CO_2_ was calculated as evolved CO_2_–C per day divided by MBC.

Ergosterol was measured according to Djajakirana et al.^40^: 2 g soil was extracted with 100 ml ethanol by oscillated shaking at 250 rev min^−1^ for 30 min. Ergosterol in the extracts was detected at a wavelength of 282 nm during reverse-phase HPLC with 100% methanol as the mobile phase^40^.

### Statistical analysis

Prior to statistical analyses data was cleaned for outliers by winsorizing with mean + /− one standard deviation. Normal distribution of residuals was tested by the Shapiro–Wilk test and QQ plots, and the homogeneity of variances by the Levene test. In case of non-normal distribution of residuals, data was transformed with an exponential function to meet the assumptions for a univariate analysis of variance (ANOVA). All parameters were tested by a univariate ANOVA with block and sheep density as fixed factors and without interactions of the two factors. At a significance level of p < 0.05 Tukey HSD tests were conducted post-hoc. To test non-linear correlations between measured parameters Spearman rank correlation coefficients were calculated. All statistical tests were conducted using SPSS 24 (IBM Deutschland GmbH, Ehningen, Germany). Results shown in the tables are arithmetic means (n = 4) and refer to an oven-dry basis (105 °C, 24 h). The coefficient of variation values (CV %) represent the mean CV across the grazing treatments.

## Results

The texture of the rangeland soil consisted on average of 36% sand, 41% silt, and 23% clay. The mean soil pH was 6.0 and electric conductivity 296 µS cm^−1^ (Table 1). Statistical analyses of these parameters did not reveal any statistically significant effect of stocking densities on these parameters (p > 0.29). Water holding capacity (WHC), SOC, and total N were relatively high with on average 76%, 38 mg g^−1^, and 3.6 mg g^−1^, respectively, and peaked, though not significantly, at a sheep density of 8 sheep ha^−1^ (Table 2). The soil C/N ratio was remarkably constant around 10.4.

**Table 1:** Soil texture, pH and electric conductivity (EC) of a rangeland soil grazed by 0, 8, 16, and 24 sheep ha^−1^ in a two year grazing experiment from 2014–2015 on a summer pasture in the Chinese Altai Mountains, Qinghe, China.

Stocking density	Sand	Silt	Clay	pH	EC_1:2.5_
sheep ha^*−*1^	%				µS cm^*−*1^
0	34.9	41.7	23.3	6	396.6
8	34.9	41.5	23.6	6.1	285.4
16	36.1	41.5	22.5	6	299.4
24	37.4	40.6	21.8	6	303.4
CV (%)	8.5	7.9	5.3	3.7	14.2
p-value stocking density	0.76	0.93	0.29	0.93	0.95
p-value block	0.51	0.34	0.25	0.87	0.25

CV = mean coefficient of variation for grazing treatment.

**Table 2:** Water holding capacity (WHC), SOC, total N and soil C/N ratio of a rangeland soil grazed by 0, 8, 16, and 24 sheep ha^−1^ in a two year grazing experiment from 2014–2015 on a summer pasture in the Chinese Altai Mountains, Qinghe, China.

Stocking density	WHC	SOC	Total N	Soil C/N
sheep ha^*−*1^	%	mg g^*−*1^ soil		
0	77.4	38.7	3.57	10.4
8	79.1	39.2	3.73	10.5
16	75.5	37.6	3.62	10.3
24	75.1	37.4	3.59	10.4
CV (%)	4.4	13.5	8.8	0.9
p-value stocking density	0.36	0.91	0.94	0.37
p-value block	0.03	0.08	0.11	0.57

**Figure 1:**
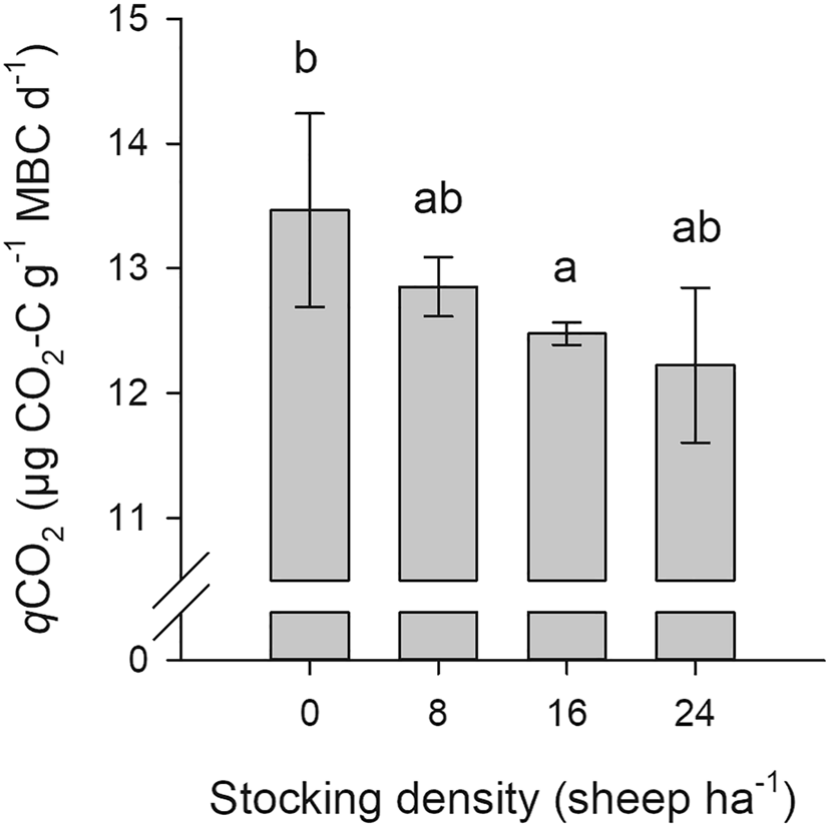
Mean metabolic quotient (*q*CO_2_) with standard error of means of a rangeland soil grazed by 0, 8, 16, and 24 sheep ha^−1^ in a two year grazing experiment from 2014–2015 on a summer pasture in the Chinese Altai Mountains, Qinghe, China. Data was transformed with an exponential function to obtain normal distribution of residuals. Letters indicate significant differences between treatment means (p < 0.05).

Mean MBC was 831 µg g^−1^ soil and MBN 134 µg g^−1^ soil and contributed on average 2.2% to SOC and 3.7% to total N (Table 3). The MB-C/N ratio varied slightly around 6.2 across all sheep densities. Microbial biomass responded to grazing pressure with lowest values at 16 sheep ha^−1^ and peaks at 24 sheep ha^−1^. Ergosterol content and basal respiration followed a similar trend as SOC with highest values at a stocking density of 8 sheep ha^−1^ (6.0 µg g^−1^ soil and 10.7 µg CO_2_–C g^−1^ MBC d^−1^, respectively). Ergosterol contributed with strong variations on average 0.66% to MBC. The *q*CO_2_ statistically significantly decreased with increasing sheep density by in total 9% (p = 0.04; Fig. 1). The statistical analysis revealed significant effects of blocks (p values ranged from 0.02 to 0.04) for WHC, MBC, ergosterol and basal respiration, indicating a high spatial heterogeneity of the experimental plot, which considerably varied even within blocks (Table 3).

**Table 3:** Soil microbiological properties of a rangeland soil grazed by 0, 8, 16, and 24 sheep ha^−1^ in a two year grazing experiment from 2014–2015 on a summer pasture in the Chinese Altai Mountains, Qinghe, China.

Stocking density	Microbial biomass					Ergosterol		CO_2_–C
	C		N		C/N			
sheep ha^*−*1^	µg g^*−*1^ soil	% SOC	µg g^*−*1^ soil	% total N		µg g^*−*1^ soil	% MBC	µg g^*−*1^ soil d^*−*1^
0	840.7	2.2	131.7	3.69	6.13	5.8	0.67	10.4
8	832.1	2.19	135.6	3.64	6.2	6.01	0.69	10.7
16	801.5	2.13	130.2	3.58	6.18	5.64	0.63	10
24	851.2	2.22	137.1	3.84	6.24	5.48	0.65	9.4
CV (%)	15.8	4.8	9.3	3.2	2.1	22.1	8.7	13.7
p-value stocking density	0.87	0.07	0.9	0.05	0.94	0.88	0.72	0.57
p-value block	0.04	0.02	0.1	0.15	0.83	0.03	0.32	0.02

**Figure 2:**
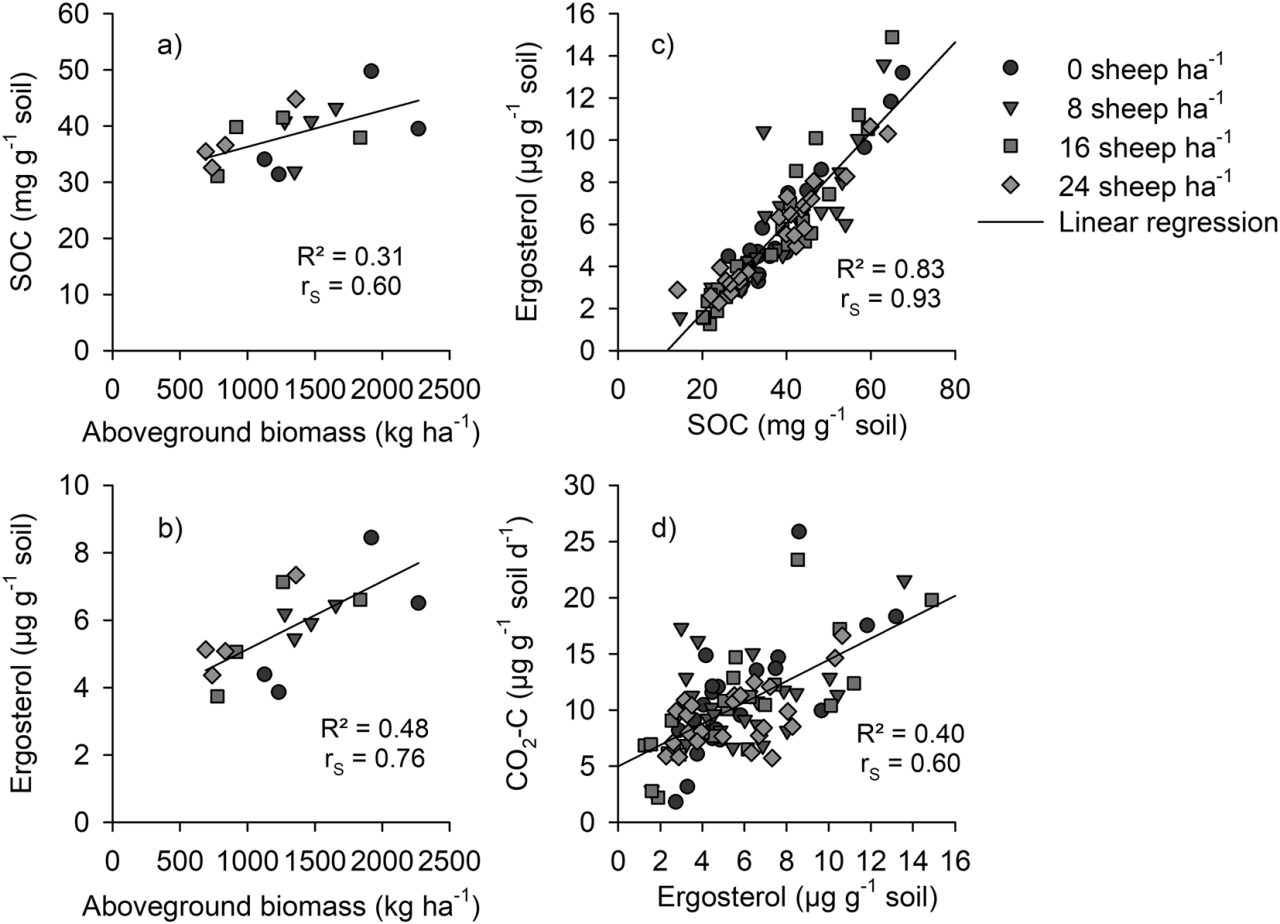
Correlations between aboveground biomass and SOC (**a**, n = 16), aboveground biomass and ergosterol (**b**, n = 16), SOC and ergosterol (**c**, n = 96), and ergosterol and basal respiration (**d**, n = 96) of a rangeland soil across all grazing intensities (0, 8, 16, and 24 sheep ha^−1^) at the end of a two year grazing experiment from 2014–2015 on a summer pasture in the Chinese Altai Mountains, Qinghe, China.

Spearman rank correlation coefficients revealed a positive relationship between SOC and AGB (r_s_ = 0.60; Fig. 2a). Additionally, statistically significant positive correlations between SOC and the biological soil properties MBC, ergosterol (Fig. 2c), and basal respiration (r_s_ = 0.60–0.93) were observed. Furthermore, MBC showed a statistically significant positive correlation with basal respiration (r_s_ = 0.51), whereas the latter positively correlated with AGB (r_s_ = 0.66). Ergosterol revealed a positive relationship with basal respiration too (r_s_ = 0.60; Fig. 2d), but additionally with the soil C/N ratio (r_s_ = 0.53) and AGB (r_s_ = 0.76, Fig. 2b).

## Discussion

In recent decades, livestock numbers and thus stocking densities of Chinese grasslands have increased as a result of the rising demand for livestock products^9,10^. Such increases in the stocking density can lead to a decrease in AGB residues^31,41,42^ and in belowground biomass^22,23^ at high grazing intensities. The lower quantity of plant biomass results in less C input into the soil by AGB detritus, root biomass, and root exudates^43,44^, which is reflected in a decrease in SOC with rising grazing intensities not only in the present study but also at other locations in Chinese grasslands^23^.

A lower root-derived organic input is generally accompanied by reduced basal respiration, which indicates C limitation and starvation of soil microorganisms^45^. Soil microorganisms are able to switch to a dormant metabolic state, when substrate availability is limited^45^. Under starvation the metabolic quotient (*q*CO_2_) is reduced, indicating an aged microbial community^46,47^. A dormant and aged microbial biomass diverts less SOC to catabolic physiological processes within the cell, referring to the reduced demand for maintenance energy^45–47^. This reduced demand for maintenance energy enables an aged microbial community to survive long periods under C limiting conditions. As observed in this study, grazing at higher stocking densities resulted in a lower *q*CO_2_ accompanied by reduced SOC accumulation due to a reduced organic input by removing AGB^12,21^, which limits the energy supply for soil microorganisms.

In the long-term MBC was found to decline with high sheep stocking rates^43,48^. In the short-term, i.e. within the two years of the present study, the high sheep stocking density did not significantly reduce MBC due to the strong survival strategies of the Chinese grassland microbial communities. The turnover of microbial biomass is generally slow due to the extremely low temperatures in winter and long periods of drought in summer^49^, which allows a long survival under C limited conditions^45^. This slows down the microbial response to intensive sheep grazing of AGB.

The spatial variability of soil properties is generally high in grassland systems of the Altai Mountains^50^. Additionally, animal grazing creates a considerable heterogeneous spatial distribution of C and nutrients in the topsoil by the punctual excretion of urine and discharge of feces^21,24^, creating hot spots of microbial biomass and activity in the field^51,52^. Furthermore, the mechanical impact of livestock hooves during treading lead to a spatial variability of soil properties by promoting soil compaction, breakdown of soil aggregates and incorporation of plant litter into the soil, which can either inhibit or accelerate microbial growth^21,28^. The stocking density thereby determines the extent of spatial variability^21,53^. Therefore, the ANOVA results were in most cases not significant in contrast to the Spearman correlation coefficients. For the same reason of spatial variability, there was no significant effect of stocking density on soil C and N. The AGB, however, which was affected by grazing intensity^31^, correlated with SOC as previously found elsewhere on Chinese grasslands^23^.

The concentration of the cell membrane component ergosterol in the soil, an indicator for the biomass of saprotrophic fungi, correlated with the AGB and even more strongly with the SOC concentration, which is of pivotal importance for microorganism as a food source^25,26,44^. The ergosterol concentration and its contribution to the total microbial biomass was about four times higher than in rangeland soils of a Mongolian river oasis in the Altai Mountains^54^, but within the range reported for Swiss subalpine pastures^28^. The correlation of ergosterol with basal respiration underlines the importance of fungi for humus mineralization of this steppe soil.

The highest values not only for ergosterol but also for SOC and basal respiration were observed at a low stocking density of 8 sheep ha^−1^. There is considerable evidence that the belowground biomass and the root C pool, respectively, increase under low or moderate stocking densities compared to zero grazing, as shown not only for alpine grasslands^42^ but also for global grasslands^43^. Additionally, it has been observed that grazing increases root exudation rates in grasslands shortly after grazing events^43, 55^ as herbivory delays or prevents the annual maturation of plants leading to root exudation of labile C-containing components^56^. As a food source, labile C stimulates soil biota. This was shown for a soil of a grazed alpine meadow in the Sichuan province, China, where relatively strong correlations between root exudation rate and microbial biomass as well as microbial activity were observed^44^. In the present study, increasing the stocking density from 0 to 8 sheep ha^−1^ therefore most likely resulted in an increased allocation of photosynthetically fixed C to the root zone (in form of root C and root exudations), which was reflected by the observed higher SOC values. The stimulation of soil microorganism through the high root-derived organic inputs leads to the high basal respiration rates and the high portion of saprotrophic fungi, which are of particular importance for humus mineralization in this steppe soil, as discussed before. Additionally, it should be noted that there are possibly other maxima of soil biological parameters between stocking densities of 0 and 8 sheep ha^−1^ or 8 and 16 sheep ha^−1^. Only a follow-up study with more grazing intensities (e.g. 0, 4, 8, 12, 16 sheep ha^−1^) could provide evidence on further maxima.

## Conclusions

Though ABG was significantly reduced by increasing stocking density, the analyzed soil parameters of the subalpine steppe in the Chinese Altai Mountains were in general not significantly affected after two years of treatment implementation, due to a high spatial variability caused by the grazing animals. Only the metabolic quotient (*q*CO_2_), which proved to be a sensitive indicator of soil degradation even after two seasons, significantly declined with increasing stocking density, indicating an aged microbial biomass, likely starving under C limitation. The effect of stocking density on soil fertility was indirect, as soil fertility parameters such as SOC and ergosterol correlated with AGB, which significantly declined with increasing stocking density. Like *q*CO_2,_ SOC, ergosterol and basal respiration also were highest at 8 sheep ha^−1^ indicating that a modest stocking density is recommendable to avoid soil degradation of the subalpine steppe. A follow-up study with stocking densities between 0 and 16 sheep ha^−1^ (e.g. 0, 4, 8, 12, 16 sheep ha^−1^) could provide indications about an optimal stocking density.
